# Utility of asymptomatic inpatient testing for COVID-19 in a low-prevalence setting: A multicenter point-prevalence study

**DOI:** 10.1017/ice.2020.349

**Published:** 2020-07-22

**Authors:** Anthony D. Bai, Xena X. Li, Mohammed Alsalem, Sarah Khan, Marek Smieja, Dominik Mertz, Zain Chagla

**Affiliations:** 1 Division of Infectious Diseases, McMaster University, Hamilton, Ontario, Canada; 2 Infection Prevention Control, McMaster Children’s Hospital, Hamilton, Ontario, Canada; 3 Laboratory Medicine, St Joseph’s Healthcare Hamilton and Hamilton Health Sciences, Hamilton, Ontario, Canada; 4 Infection Control, Hamilton Health Sciences, Hamilton, Ontario, Canada; 5 Infection Control, St. Joseph’s Healthcare Hamilton, Hamilton, Ontario, Canada

For coronavirus disease 2019 (COVID-19), frequently reported symptoms in nonseverely sick patients include fever, fatigue, and dry cough.^1^ However, infected patients may not exhibit symptoms. Some patients may be presymptomatic and develop symptoms later in the disease course whereas others remain asymptomatic, but either group can be infectious.^2,3^

Hence, asymptomatic carriers and presymptomatic individuals may be potential sources of nosocomial transmission. As such, consideration can be given to testing asymptomatic patients upon admission to the hospital. The Infectious Diseases Society of America (IDSA) guidelines on the diagnosis of COVID-19 recommend against testing of asymptomatic hospitalized patients in low-prevalence (<2%) settings.^4^ This recommendation is based on expert opinion and lacks supporting evidence.

The city of Hamilton, Ontario, Canada, has a population of 580,000 and qualified as a low-prevalence area at the time of this study. The average number of daily new cases identified was 1.9 per 100,000 population.^5^ For hospital admission, the testing strategy was (and continues to be) based on symptoms or exposures.^6^ Within this low-prevalence setting, we conducted a multicenter point-prevalence study to evaluate the utility of severe acute respiratory coronavirus virus 2 (SARS-CoV-2) testing of asymptomatic patients in terms of capturing positive cases that would be missed by symptom-based testing on admission.

## Methods

We conducted a point-prevalence study across 4 tertiary acute-care hospitals in Hamilton from April 15 to April 21, 2020. The Hamilton Integrated Research Ethics Board approved this study (no. 10894).

### COVID-19 testing on admission

According to provincial guidelines, testing was based on the following symptoms: fever, new or worsening acute respiratory illness symptom (ie, cough, dyspnea, sore throat, runny nose or sneezing, nasal congestion, hoarse voice, difficulty swallowing, new olfactory or taste disorder(s), nausea or vomiting, diarrhea, abdominal pain), or clinical or radiological evidence of pneumonia.^6^ Atypical presentations included unexplained fatigue or malaise, delirium, falls, acute functional decline, exacerbation of chronic conditions, chills, headache, croup, tachycardia, decrease in blood pressure, hypoxia, and lethargy.^6^ At the time of this study, a patient with any of the above symptoms or exposure underwent nasopharyngeal swab testing for SARS-CoV-2 upon admission to the hospital.^6^

### Patient inclusion

On the point-prevalence testing date, all adult inpatients were tested once if they were admitted for 7–14 days, regardless of symptoms or prior negative SARS-CoV-2 test result. Patients with a known positive SARS-CoV-2 test were excluded.

Testing on days 7–14 was based on the estimated median incubation period of 4 days (interquartile range, 2–7 days).^7^ Testing after the median incubation period would have captured most COVID-19 cases, even if the exposure occurred as late as the day of admission.

### Testing procedure

The nasopharyngeal swabs were collected, and a polymerase-chain reaction assay for the SARS-CoV-2 envelope and 5’-untranslated region genes was performed at the local virology laboratory in the hospital. This assay was validated against the provincial standard testing.

### Data collection

Data were extracted from the patient electronic chart system, which included demographics, admitting diagnosis, hospital location, admitting service, reason for admission, Charlson comorbidity index,^8^ prior SARS-CoV-2 test result, chest imaging, and other microbiology test results. On the day of testing, patients were assessed for symptoms, as listed above.^6^

## Results

Across the 4 hospitals, 125 inpatients were tested for SARS-CoV-2 (Table 1). Also, 5 patients (4.0%) had fever and 3 patients (2.4%) had respiratory symptoms at the time of their test.

**Table 1.** Patient Characteristics

Characteristic	Patients Tested (N = 125), No. (%)^a^
**Demographics**	
Age, median y (IQR)	77.0 (65.0–84.0)
Male	58 (46.4)
**Location prior to admission**	
Community	94 (75.2)
Hospital	16 (12.8)
Retirement home	11 (8.8)
Long term care home	4 (3.2)
**Hospitals^b^**	
A	52 (41.6)
B	30 (24.0)
C	29 (23.2)
D	14 (11.2)
**Location within hospital**	
Ward	95 (76.0)
ICU	8 (6.4)
Rehabilitation, palliative, or chronic care	22 (17.6)
**Admitting service**	
Medicine	49 (39.2)
Surgery	30 (24.0)
Hematology oncology, medical oncology	16 (12.8)
ICU	8 (6.4)
Obstetrics	1 (0.8)
Other	21 (16.8)
**Most common reason for admission**	
Hip fracture	11 (8.8)
Solid tumor–related complication	11 (8.8)
Stroke	7 (5.6)
Weakness or fall	7 (5.6)
Delirium or confusion	7 (5.6)
**Charlson comorbidity index**	
0	18 (14.4)
1	15 (12.0)
2	28 (22.4)
3	18 (14.4)
≥4	46 (36.8)
Immunosuppressed^c^	21 (16.8)
**Symptoms at testing**	
Fever	5 (4.0)
Respiratory symptoms	3 (2.4)
Clinical or radiographic features of pneumonia	14 (11.2)
Atypical symptoms	10 (8.0%)
**Chest imaging**	
Chest X-ray	92 (73.6)
CT chest image	26 (20.8)
**Last chest imaging findings**	
Consolidation	34 (27.2)
Intralobular lines	1 (0.8)
Organizing pneumonia	0 (0)
Days from admission to testing, median (IQR)	11.0 (9.0–13.0)
**SARS-CoV-2 test result**	
Negative	124 (99.2)
Positive	1 (0.8)
Prior SARS-CoV-2 testing done and negative	85 (68.0)
Days from prior SARS-CoV-2 test to current test	10.0 (8.0–12.0)
**Testing for other respiratory viruses^d^**	
Negative	86 (68.8)
Positive	0 (0)
Not done	39 (31.2)

Note. IQR, interquartile range; ICU, intensive care unit; CT, computed tomography.

a Units unless otherwise specified.

b The hospital sites include a 607-bed hospital that is the regional cardiac surgery and neurosurgery center; a 228-bed hospital that is the regional center for cancer care and bone marrow transplant; a 250-bed hospital that specializes in chronic care and rehabilitation; and a 426-bed hospital that specializes in dialysis and renal transplant.

c Immunosuppression includes any of the following: chemotherapy, steroid therapy, neutropenia with absolute neutrophil count <0.5, active hematological malignancy, HIV, primary immunodeficiency, or solid organ or hematopoietic stem cell transplant requiring immunosuppressive therapy.

d Other respiratory viruses include influenza A and B, respiratory syncytial virus (RSV), human metapneumovirus, parainfluenza virus types 1 and 3, adenovirus, rhinovirus, and enterovirus.

Only 1 patient (0.8%) was positive for SARS-CoV-2. This patient presented to hospital C reporting 2 weeks of fever and cough. A chest x-ray showed an ill-defined opacity in the left lower lobe. The patient was initially isolated for acute respiratory illness. Isolation was discontinued on admission day 2 after the SARS-CoV-2 test came back negative, and the patient was treated for a presumptive bacterial pneumonia. The following day, the patient was transferred to hospital D. The positive point-prevalence test occurred on admission day 13 at hospital D. At the time of testing, the patient had no new symptoms and the patient’s respiratory status continued to improve.

## Discussion

In this point-prevalence study, 125 inpatients were tested, and only 1 patient (0.8%) was positive for SARS-CoV-2. This positive case was symptomatic, and the patient had had a prior SARS-CoV-2 test that was likely a false negative. He was initially isolated for acute respiratory illness on presentation to the hospital. For COVID-19, infectiousness has been estimated to decline quickly within 7 days,^9^ so the patient was likely no longer infectious at the time of the second test. Therefore, asymptomatic testing did not add any useful information or change infection control practices compared to symptom-based screening.

To our knowledge, this is the first study to evaluate the benefit of asymptomatic testing for hospitalized patients in a low-prevalence setting. In a New York hospital, universal testing of women admitted for delivery showed 13.5% asymptomatic positive SARS-CoV-2 results.^10^ In contrast, our study found no asymptomatic positive cases. This finding is likely due to differences in local prevalence.

The strengths of our study include the systematic approach to testing. Also, the inclusion of 4 hospitals makes the results more generalizable. Our study has 2 limitations. First, repeated point-prevalence testing would have yielded more precise results, but this method would not have been feasible given the capacity of our virology laboratory. Second, nasopharyngeal swabbing may produce false-negative results, given its estimated sensitivity between 75% and 95%.^4^ Although imperfect, nasopharyngeal swabbing is practical and is currently the recommended test for asymptomatic patients.^4^

In conclusion, our study suggests the minimal utility of asymptomatic testing in hospitalized patients compared to symptom screening and targeted testing in low-prevalence settings, which supports the current IDSA guidelines.^4^
